# Shame and honour drive cooperation

**DOI:** 10.1098/rsbl.2011.0367

**Published:** 2011-06-01

**Authors:** Jennifer Jacquet, Christoph Hauert, Arne Traulsen, Manfred Milinski

**Affiliations:** 1 Department of Mathematics, University of British Columbia, 1984 Mathematics Road, Vancouver, British Columbia, CanadaV6T 1Z2; 2 Sea Around Us Project, University of British Columbia, 2202 Main Mall, Vancouver, British Columbia, CanadaV6T 1Z4; 3 Research Group for Evolutionary Theory, Max Planck Institute for Evolutionary Biology, August-Thienemann-Strasse 2, 24306 Ploen, Germany; 4 Department of Evolutionary Ecology, Max Planck Institute for Evolutionary Biology, August-Thienemann-Strasse 2, 24306 Ploen, Germany

**Keywords:** cooperation, honour, shame, public goods game, tragedy of the commons

## Abstract

Can the threat of being shamed or the prospect of being honoured lead to greater cooperation? We test this hypothesis with anonymous six-player public goods experiments, an experimental paradigm used to investigate problems related to overusing common resources. We instructed the players that the two individuals who were least generous after 10 rounds would be exposed to the group. As the natural antithesis, we also test the effects of honour by revealing the identities of the two players who were most generous. The non-monetary, reputational effects induced by shame and honour each led to approximately 50 per cent higher donations to the public good when compared with the control, demonstrating that both shame and honour can drive cooperation and can help alleviate the tragedy of the commons.

## 1. Introduction

Shame is a traditional deterrent from asocial behaviour and is employed when offenders are singled out for public scorn. The expectation of honour, on the other hand, can reinforce prosociality. While honour is conspicuous in society (e.g. the proliferation of prizes), it is tempting to think of shame as a medieval phenomenon, when the accused were placed in the town pillory or emblazoned with a scarlet letter. Modern democratic societies have moved away from including the public in the punishment, although in some cases (e.g. drunk driving licence plates) the state still sanctions shame [1]. Furthermore, attention in the form of shame as well as honour could become more prevalent as digital technology increasingly allows us to communicate and keep track of one another. Here, we test experimentally whether the fear of being shamed or the prospect of being honoured provides an incentive to cooperate.

Social dilemmas arise through the consumption of common resources, such as wild fish, fossil fuels or clean water, and translate into a tragedy of the commons, where group cooperation is undermined by individual self-interest [2]. Public goods experiments capture the tension between individual and group-interest, and usually confirm Hardin's pessimistic promise that ‘freedom in the commons brings ruin to all’ [3]. In a typical set-up, players receive start-up capital and can choose to donate some or none of it to a ‘public goods’ project; donations are increased by a given factor and redistributed evenly among all players, irrespective of whether they contributed. Maximum net benefit is achieved if all players donate, but individual players earn most if they keep their capital and profit from the generosity of the other players. Usually players inevitably exercise this ‘rational’ self-interest and cooperation quickly declines.

Public goods interactions also exemplify cooperation's intricacies. For instance, players are willing to pay to punish uncooperative behaviour [4]. Experiments that allow players to build and benefit monetarily from reputation lead to increased cooperation [5,6]. In games that offer players anonymity, uncooperative behaviour is more prevalent [7] while the opposite is true of games in which players know that each of their decisions will be linked to their real identities [8–10]. Revealing the identities of all participants (e.g. [8–10]) corresponds to full transparency but publicizing all identities does not allow us to distinguish whether increased cooperation is primarily due to the promise to expose low contributors, high contributors or both. If players know that only the least or most cooperative individuals are to stand in front of their peers, will they cooperate more as a group?

We designed this public goods experiment to isolate the effects of being shamed or honoured, with no monetary consequences to either experience, and test whether the expectation of negative or positive reputational information enforces social behaviour. We hypothesized that the threat of shame or the prospect of honour would lead to increased public contributions. We also expected that shame might be more effective than honour because players would particularly seek to avoid negative exposure, and therefore contribute more to the public good.

## 2. Material and methods

We tested our predictions with 180 first-year University of British Columbia science students divided into three treatments, shame, honour and control, consisting of 10 identical six-player games each. To foster indelibility of being shamed and honoured, all six players came from the same class so that the players were acquainted with each other. Players were recruited within the first few weeks of the term to ensure that they would meet again repeatedly during the term.

There was a single group of six players in the room at a time. Players were partitioned off from each other as well as the experimenters, who stayed out of view for the duration of the actual experiment. Each player received a starting account of CDN$12 and a randomly assigned unique pseudonym (obscure Greek gods). Players were anonymized, both to the experimenters and other players, but players in the honour and shame treatments wrote real names inside an envelope labelled with their pseudonym, which was collected by the experimenter so the two least generous players (or most generous in the honour treatment) could eventually be identified. The box with the concealed names remained visible to all players at all times to protect their anonymity. All six players could see a public screen on which instructions and the game were projected. Before the game, an experimenter read the instructions, and demonstrated the choices and outcomes in example games using pseudonyms not appearing in the experiment.

Players chose whether to contribute $1 into a public pool or keep it in his/her private funds at each round for 12 rounds. Without visual contact with the player, an experimenter passed a locked box into each cubicle, in which every player placed his/her anonymized envelope (blank on the outside; pseudonym on the inside) containing $0 or $1. Contributions were recorded on the public screen under each player's pseudonym. The group total and player payout were displayed for each round, as was the aggregate total contribution for each player.

After round 10, the experimenter opened the envelopes labelled with the pseudonyms of the two players who donated least overall in the shame treatments to reveal their real names (in the honour treatment it was the two players who donated most). In the event of a tie, the experimenter chose two players by throwing a six-sided die, with the pseudonyms pre-determinedly linked to each number. Ties occurred in five of the shame games and four of the honour games. Interestingly, ties occurred only for the second least (or most) generous players but never for the least (or most) generous players. In addition, one game in the shame treatment resulted in only one player being exposed because all five other players contributed 100 per cent. The two least generous players went in front of the group and wrote their name on a board under the phrase ‘I donated least’, which was visible for the entire game (for honour, the phrase was ‘I donated most’ and the two most generous players went in front). The real names of these two players were also added to the pseudonyms on the public screen. The remaining four envelopes with the names of the four players that retained their anonymity were visibly destroyed and discarded in front of the group. In the control treatment, all six players remained anonymous. At the end of round 12, each player left with the money he/she kept during the game plus the profits from the public pool. Note that the profits from the public pool were the same for every player and could therefore be distributed without compromising the players' anonymity. The students were asked not to discuss the experiment with anyone else.

## 3. Results

**Figure 1.**
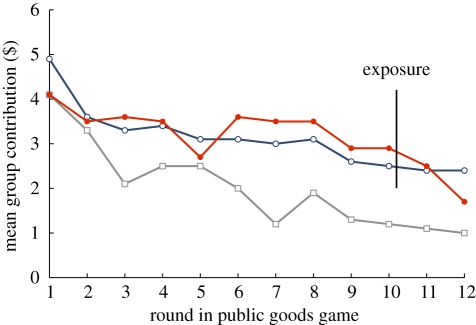
Average group contributions for each treatment: shame (red with filled circles), honour (blue with open circles) and control (grey with open squares). In the shame treatment, the two least generous players were exposed as free riders after round 10 while in honour the two most generous were revealed as highest contributors to the group. In the control treatment, all players retained anonymity over the 12 rounds, as did the non-exposed players in shame and honour.

In each treatment, initial cooperation in the public goods game declined as expected (paired *t*-test between 1st and 10th round, *n*_s_ = 10, *t* = 2.71, *p* = 0.024; *n*_h_ = 10, *t* = 4.61, *p* = 0.001; *n*_c_ = 10, *t* = 7.61, *p* < 0.0001; the six-player group is the statistical unit unless otherwise noted; all statistical tests are two-tailed; figure 1). Donations for the first 10 rounds in the shame treatment were significantly higher when compared with the control (two-sample *t*-test, *n*_s_ = 10, *n*_c_ = 10, *t* = 2.24, *p* = 0.038), as were contributions in the honour treatment (two-sample *t*-test, *n*_h_ = 10, *n*_c_ = 10, *t* = 2.89, *p* = 0.010). Average group contributions were 53 per cent higher in the shame treatment ($33.8 ± $13.6 s.d.) and 48 per cent higher in honour ($32.6 ± $6.6 s.d.) than in the control ($22.1 ± $9.4 s.d.; full cooperation is $60 in donations).

In contrast to our expectations, we found no significant differences in group contributions over the first 10 rounds between the shame and honour treatments. More surprising, contributions from the least generous ‘shamed’ players ($3 average) were not significantly different from the anonymous, least generous contributors in the honour treatment ($2.4 average), nor were contributions from the most generous ‘honoured’ players ($8 average) significantly different from the anonymous, most generous players' contributions in the shame treatment ($7.7 average; electronic supplementary material, figure S1). The threat of being shamed does not work only to encourage cooperation from the least cooperative players, nor does the promise of honour work to encourage cooperation only from the most cooperative players. Rather, both treatments led to a general increase in average player contribution, confirming that, even when only the least or most cooperative individuals are to stand in front of their peers, players cooperate more as a group (electronic supplementary material, figure S1).

Our results show that a promise to single out free-riding individuals for public scrutiny can lead to greater cooperation from the whole group, as can singling out the most generous individuals. Even in this one-off experiment, people were willing to pay, not necessarily to avoid exposure, but to avoid being shamed, and thereby avoid a potential loss of reputation within their social sphere. Group cooperation in the shame treatment significantly declined following round 10 (paired *t*-test between 10th and 12th round, *t* = 3.67, *p* = 0.005), corroborating our finding that the threat of being singled out as a free rider had been driving cooperation. By contrast, players in the honour treatment did not fear exposure; they paid for it. Furthermore, cooperation in the honour treatment was maintained in rounds 11 and 12 possibly because once players earned their honorable reputation they felt obliged to maintain it. Average contributions from anonymous players in round 12 were very similar in the treatments for honour ($0.33) and shame ($0.34), but average contributions from players who were honoured ($0.55) were significantly higher than those from players who were shamed ($0.15; two-sample *t*-test, *n*_h_ = 20, *n*_s_ = 19, *t* = 2.72, *p* = 0.009; the individual player is the statistical unit).

## 4. Discussion

Cues of being watched enhance cooperation [11] and when humans lived in small groups, it was easy to observe individual behaviour. However, as human society grew, gossip, by way of language, replaced direct observation as a vector for keeping track of human behaviour [12,13]. At this transition, shame and honour could have been at a premium—when the chance of witnessing behaviour firsthand was then amplified by the possibility that it could be verbally expressed to the community.

Shame is an uncomfortable phenomenon, in part, because it invites the public to join in the punishment. Today, there are also convincing philosophical objections to a legal system that shames individuals on the grounds that such punishments undermine human dignity [1]. But the absence of shaming by the state does not preclude the absence of shame altogether in society, especially as social media increases the frequency, speed and inclusiveness of communication. The Internet increasingly creates a global town square where controls are harder to implement and enforce, gossip travels fast, and where shame as well as honour therefore might experience resurgence. At the same time, the Internet is also a tool for tracking compliance and for transparency (e.g. [14]). Transparency also enhances cooperation [8–10] but can be costly to provide and its use can be limited. Transparency requires time evaluating and determining a satisfactory performance. This becomes increasingly difficult in our current era, where human attention, not information, is a scarce resource [15]. By singling out only the least or most cooperative players, attention in the form of shame or honour may be more parsimonious than full transparency and relies on social norms as reference points.

In this experiment, the fear of being shamed as well as the promise of being honoured led to increased cooperation from the entire group and might even help transform a crowd into a community. The results reinforce honour as a motivation to cooperate and also illuminate a potential positive consequence in the unavoidable revival of the old threat of shame: to encourage groups to maintain resources that we all share.
